# The efficacy and safety of Chinese herbal compound in pediatric patients with allergic rhinitis: A protocol for systematic review and meta-analysis

**DOI:** 10.1097/MD.0000000000021643

**Published:** 2020-08-07

**Authors:** Mengni Zhang, Yihua Fan, Chunying Tian, Yue Xie, Yao Huang, Shasha Yang, Qinxiu Zhang

**Affiliations:** a Chengdu University of Traditional Chinese Medicine; b Hospital of Chengdu University of Traditional Chinese Medicine, Chengdu, Sichuan province; c Tianjin University of Traditional Chinese Medicine, Tianjin; d First Affiliated Hospital of Guizhou University of Traditional Chinese Medicine, Guiyang, Guizhou province; e School of Medical and Life Sciences/Reproductive & Women-Children Hospital, Chengdu University of Traditional Chinese Medicine, Chengdu, China.

**Keywords:** Chinese herbal compound, pediatric, allergic rhinitis, systematic review

## Abstract

**Background:**

We design this study to assess the efficacy and safety of Chinese herbal compound for allergic rhinitis in children.

**Methods:**

PubMed, EMbase, Cochrane Library, Web of Science, China National Knowledge Infrastructure (CNKI), WanFang, the Chongqing VIP Chinese Science and Technology Periodical Database, and China biomedical literature database (CBM) will be searched from the establishment of each database to July 2020. Randomized controlled trials of Chinese herbal compound for the treatment of allergic rhinitis in children will be included. Two researchers will screen the literature, extract data, and assess the risk of bias independently. Statistical analysis will be performed in RevMan 5.3.

**Results:**

This study will summarize high quality evidence of randomized controlled trials on exploring the efficacy and safety of Chinese herbal compound for allergic rhinitis in children.

**Conclusions:**

The findings of study will provide scientific evidence of the efficacy and safety of Chinese herbal compound for allergic rhinitis in children for clinician and further studies.

**Ethics and dissemination:**

The private information from individuals will not be published. This systematic review also will not involve endangering participant rights. Ethical approval is not required. The results may be published in a peer-reviewed journal or disseminated in relevant conferences.

**OSF Registration number:**

DOI 10.17605/OSF.IO/Q5TRZ.

## 1 Introduction

Allergic rhinitis in children is a non-infectious inflammatory disease of nasal mucosa caused by exposure to allergens and mediated mainly by immunoglobulin E (IgE) in susceptible children.^[1]^ The 4 typical symptoms of allergic rhinitis in children are sneezing, watery nose, nasal itching, and nasal congestion. Infants can show nasal congestion, accompanied by mouth opening breathing, snoring, wheezing, feeding difficulties, nose, and eyes rubbing, etc.^[2]^. Allergic rhinitis has become a major respiratory inflammatory disease in children, mostly occurring between 8 and 13 years old, with an increasing incidence year by year.^[3,4]^ Due to childrens difficulty in self-management, various symptoms could not only seriously affect childrens physical and mental health, academic performance and quality of life, but also lead to various complications, including asthma, conjunctivitis, and adenoid hypertrophy.^[5,6]^ Studies have found that allergic rhinitis in children is related to allergy, low immunity, heredity, air pollution, and other factors.^[7]^ The main pathological mechanism is the entry of antigens into the sensitized individuals, which causes the release of relevant inflammatory mediators and the aggregation of inflammatory cells, thus triggering a series of symptoms.^[8]^ The treatment of allergic rhinitis in children requires the combination of prevention and treatment, which includes environmental control, drug therapy, immunotherapy, and health education.^[9,10]^ Drug therapy mainly includes glucocorticoids, antihistamines, and leukotriene receptor antagonists.^[11]^ Although those drugs can quickly relieve symptoms and slow down disease progression, it is difficult to fundamentally improve allergy, with poor long-term efficacy and high risk of recurrence. Moreover, long-term use of those drugs is prone to drug resistance and side effects in different levels (such as growth retardation, fatigue, impaired liver and kidney function, gastrointestinal reactions, etc.).^[12]^

The traditional Chinese medicine compound has the treatment characteristic of multi-pathways, multi-links and multi-targets, and has the potential prevention and treatment advantages. The treatment of allergic rhinitis in children with traditional Chinese medicine compound has the effect of regulating immunity and reducing allergic state, with the advantages of safety, reliability, stable efficacy, low recurrence rate, minimal toxicity and side effects, which has attracted wide attention.^[13]^ However, there is no systematic review and meta-analysis evaluating the efficacy and safety of Chinese herbal compound in pediatric patients with allergic rhinitis. Thus, this study will evaluate the efficacy and safety of Chinese herbal compound in pediatric patients with allergic rhinitis.

## 2 Methods

### 2.1 Study registration

This protocol of systematic review and meta-analysis has been drafted under the guidance of the preferred reporting items for systematic reviews and meta-analyses protocols (PRISMA-P). Moreover, it has been registered on open science framework (OSF) on July 8, 2020. (Registration number: DOI 10.17605/OSF.IO/Q5TRZ)

### 2.2 Ethics

Ethical approval is not required because there is no patient recruitment and personal information collection, and the data included in our study are derived from published literature.

### 2.3 Inclusion criteria for study selection

#### 2.3.1 Type of studies

Randomized controlled trials involving Chinese herbal compound for pediatric patients with allergic rhinitis will be included. The language will be limited to Chinese and English.

#### 2.3.2 Type of participants

Participants conformed to Guidelines for diagnosis and treatment of pediatric allergic rhinitis^[3]^ will be included, regardless of gender, age, and race.

#### 2.3.3 Type of interventions

The control group was treated with western medicine alone, and the treatment group was treated with Chinese herbal compound, regardless of the dosages, and duration of drug treatment.

#### 2.3.4 Type of outcome measures

The main outcome indicator was the clinical efficiency, and the efficacy was evaluated according to the score of symptoms and signs.^[14]^ Treatment index ≥66% was significant, treatment index 65% to 26% was effective, and treatment index ≤25% was ineffective. Total efficiency = (significant effective cases + effective cases)/total number of cases.

Secondary outcome measures were sign score, symptom score, IgE level, recurrence rate, and occurrence of adverse reactions.

### 2.4 Exclusion criteria

1. In addition to the traditional Chinese medicine compound, the treatment group also used other traditional Chinese medicine therapies, such as acupuncture and moxibustion, acupoint sticking, cupping, etc.;
2. Studies with unsatisfactory outcome indicators;
3. As for duplicate published literatures, select the literature with the most complete data;
4. Missing main content, unable to conduct statistical analysis, and unable to obtain the literature after contacting the author;
5. Literature with errors in random methods.

### 2.5 Search strategy

A systematic search was conducted on the randomized controlled trials of Chinese herbal compound for the treatment of allergic rhinitis in children. The databases include: PubMed, EMbase, Cochrane Library, Web of Science, China National Knowledge Infrastructure (CNKI), WanFang, the Chongqing VIP Chinese Science and Technology Periodical Database, and China biomedical literature database (CBM). The retrieval time was from the establishment of each database to July 2020. At the same time to retrieve Baidu, Google Scholar, International Clinical Trials Registry Platform (ICTRP), and Chinese Clinical Trials Registry (ChiCTR) and other databases for comprehensive literature search. The search terms were as follows: “Chinese herbal compound”, “allergic rhinitis”, “pediatric”, etc. Search strategy in PubMed is shown in Table 1.

**Table 1:**
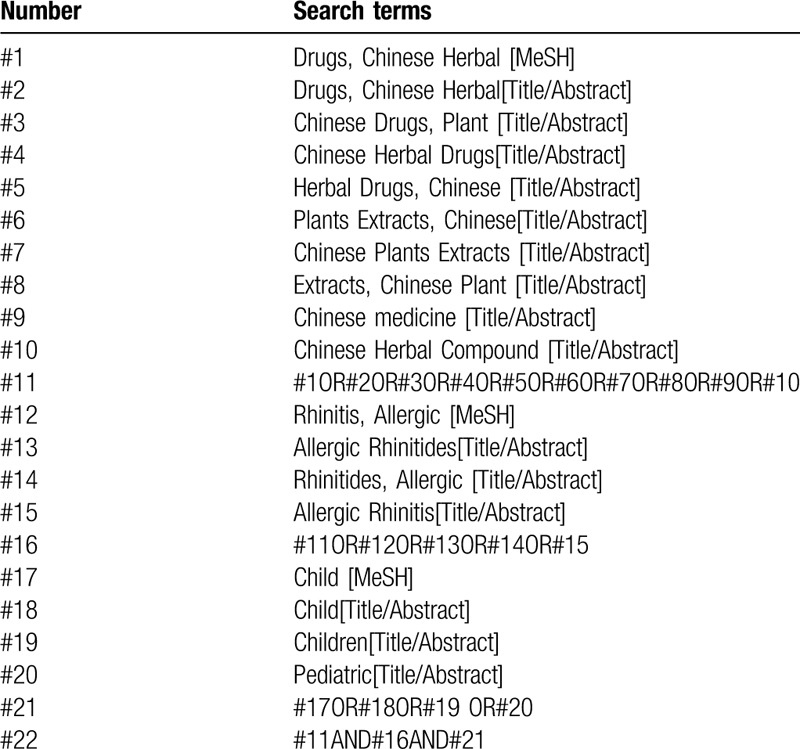
Search strategy in PubMed database.

### 2.6 Data extraction

**Figure 1:**
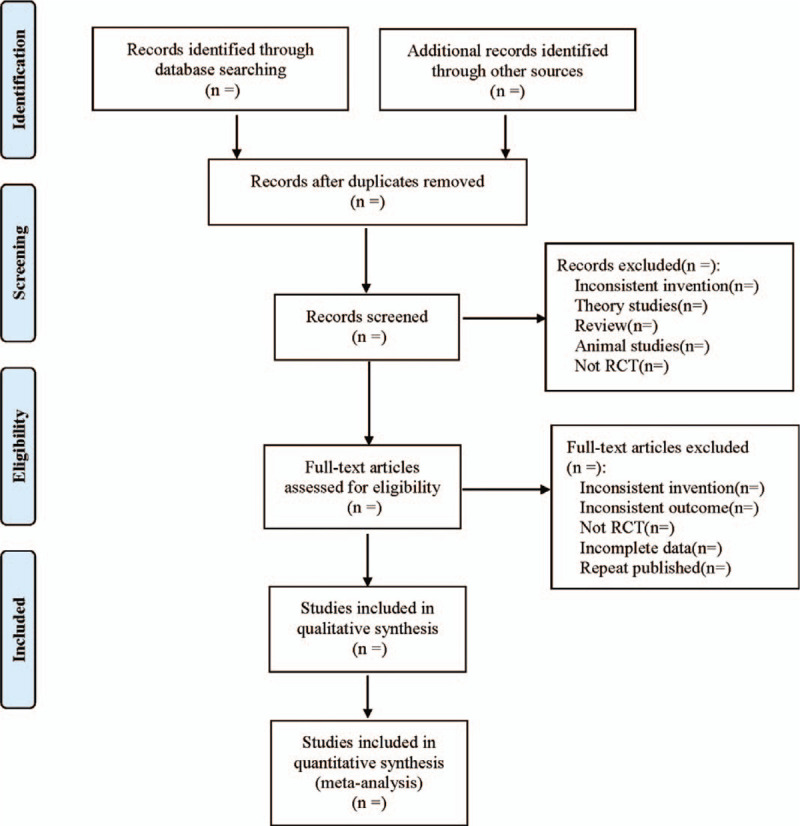
Flow diagram.

Two researchers independently screened and cross-checked the literature according to inclusion and exclusion criteria. In case of disagreement, it is resolved by discussion or with the assistance of a third investigator. Two researchers independently extracted the data, including: ① General information of the included study (title, first author, publication year, etc.); ② Baseline data of the subjects (gender, age, course of disease and degree of disease, etc.); ③ Intervention measures (name, composition, dosage, frequency and course of treatment of Chinese herbal compound; Name, dosage, frequency, and course of treatment of western medicine used in the control group); ④ The main factors in evaluating the risk of bias; ⑤ Outcome index. In case of disagreement, discuss and negotiate with the third researcher. The literature screening process is shown in Figure 1.

### 2.7 Risk of bias assessment

Two researchers independently evaluated the quality evaluation of randomized controlled trials according to the 7 dimensions of the Cochrane 5.1.0 Quality Evaluator's Manual, including: random sequence generation, allocation concealment, blinding of participants and personnel, blinding of outcome assessment, incomplete outcome data, selective reporting, and other bias. The quality of studies is classified as being at of high, unclear, or low risk of bias. In case of disagreement, discuss and negotiate with the third investigator.

### 2.8 Statistical analysis

#### 2.8.1 Data synthesis

The data were statistically analyzed using Cochrane Collaboration RevMan 5.3. The outcome index of dichotomized variables was expressed by relative risk (RR). For the outcome index of continuous variables, it was expressed by Weighted Mean Difference (WMD) if using the same measurement tools and units, and it was expressed by Standardized Mean Difference (SMD) for different measurement tools or units of data, all the above were represented by effect value and 95% confidence interval (CI). The heterogeneity between the included results was tested by χ^2^ test. If *P* ≥ .1 and *I*^*2*^ ≤ 50%, the heterogeneity was considered to be small, and the fixed-effect model was adopted. If *P* < .1 and *I*^*2*^ > 50%, inter-group heterogeneity was considered to be large, and subgroup analysis or sensitivity analysis was used to explore the source of heterogeneity. After excluding the effects of significant clinical heterogeneity, a random-effects model was used. If the inter-group heterogeneity was too large, descriptive analysis was conducted on the included literature without data merging.

#### 2.8.2 Dealing with missing data

If the data in the included study is incomplete or missing, the author will be contacted to obtain the missing data. If the data cannot be obtained, the study will be excluded from the meta-analysis.

#### 2.8.3 Subgroup analysis

In order to reduce inter-study heterogeneity and properly deal with research problems, the subjects were divided into infants and children for subgroup analysis according to their age. Subgroup analysis was conducted according to the degree of illness, course of treatment and efficacy of Chinese herbal compound.

#### 2.8.4 Sensitivity analysis

To determine the robustness of the study results, the following 3 methods were used to analyze the sensitivity of the sources of clinical heterogeneity:

1. Change the combined effect size model;
2. The study with the maximum weight removed;
3. Exclude and include the study one by one, carry out re-analysis, and observe the change of effect size and *P* value.

#### 2.8.5 Reporting bias

When more than 10 studies were included in a certain outcome indicator, funnel plot was used to determine whether publication bias existed qualitatively. Also use Egger and Begg test to quantify publication bias.

#### 2.8.6 Evidence quality evaluation

The Grading of Recommendations Assessment, Development, and Evaluation (GRADE)^[15]^ was used to evaluate the quality of evidence. Randomized controlled trials were set as the highest level of evidence, and the evaluation considers whether the quality of evidence is reduced from the following 5 factors: bias risk, consistency, directness, precision, and publication bias. And the quality of evidence will be rated as high, moderate, low, and very low.

## 3 Discussion

In traditional Chinese medicine theory, allergic rhinitis in children can be classified as the area of “BiQiu”, and the incidence is mostly related to congenital deficiencies of vital qi, dysfunction of the lungs, spleen and kidney, exogenous cold, inordinate diet, and other factors. Qi deficiency of the spleen and lung is the pathogenesis, and the treatment principles are reinforcing the spleen and lung, reducing long-standing phlegm, strengthening vital qi, and eliminating the wind.^[16]^ Huang Qi (*Radix Astragali*), Fang Feng (*Radix Saposhnikoviae*), Bai Zhu (*Rhizoma Atractylodis Macrocephalae*), Xin Yi (*Flos Magnoliae*), Cang Er Zi (*Fructus Xanthii*), Bai Zhi (*Radix Angelicae Dahuricae*), Chen Pi (*Pericarpium Citri Reticulatae*), Fu Ling (*Poria*), Jie Geng (*Radix Platycodonis*), Su Ye (*Folium Perillae*), and other traditional Chinese medicines in Chinese herbal compound are commonly used to treat allergic rhinitis in children; Xin Yi (*Flos Magnoliae*) and Cang Er Zi (*Fructus Xanthii*) can warm lung and unblock the orifices, dispel wind and cold; Chen Pi (*Pericarpium Citri Reticulatae*), Jie Geng (*Radix Platycodonis*), Su Ye (*Folium Perillae*) can warm lung, ventilate the lung qi and the middle and upper qi; Bai Zhu (*Rhizoma Atractylodis Macrocephalae*), Chen Pi (*Pericarpium Citri Reticulatae*), Fu Ling (*Poria*) can invigorate spleen and supplement qi and tonify the middle jiao, replenish body fluid of spleen; Huang Qi (*Radix Astragali*), Fang Feng (*Radix Saposhnikoviae*), Bai Zhu (*Rhizoma Atractylodis Macrocephalae*) can solidify guard qi, dispel wind without injuring vital qi.^[17]^ Experimental results ^[18]^ showed that Biminkang mixture can significantly inhibit the ileum contraction and nasal secretion exudation caused by histamine in guinea pigs. Studies have found ^[19]^ that Bishu Dropping Pills can reduce the content of serum IgE, inhibit the release of histamine in whole blood and nasal mucosa, and thus play an anti-allergic role. Clinical trials found that,^[20]^ Kebimin Spray can significantly decrease the serum IgE high values of different level in the patients with allergic rhinitis, mainly because that Fu Zi (*Radix Aconiti Lateralis Preparata*) in Kebimin Spray has adrenocorticoid-like effect, to reduce serum IgE produce, prevent being combined with target cells, to make eosinophils and mast cells in “sleep” state, to cause the loss of internal conditions for the formation of allergic rhinitis.

However, no systematic review and meta-analysis is performed to test the efficacy and safety of Chinese herbal compound in pediatric patients with allergic rhinitis. It is necessary to assess the efficacy and safety of Chinese herbal compound in pediatric patients with allergic rhinitis, to provide scientific evidence for clinician and further studies. However, the study has some limitations.

(1) The language of the literature is only Chinese and English, and there may be publication bias;

(2) Traditional Chinese medicine compound dosage form and composition are not unified, which may cause certain bias.

## Author contributions

**Data collection:** Mengni Zhang and Yihua Fan.

**Funding support:** Qinxiu Zhang.

**Literature retrieval:** Yue Xie and Chunying Tian.

**Software operating:** Yao Huang and Shasha Yang.

**Supervision:** Chunying Tian and Qinxiu Zhang.

**Writing – original draft:** Mengni Zhang and Yihua Fan.

**Writing – review & editing:** Mengni Zhang and Qinxiu Zhang.
